# Metabolic reprogramming enhances oxidative stress resistance in differentiating cardiomyocytes

**DOI:** 10.1038/s41598-026-35263-5

**Published:** 2026-01-20

**Authors:** Lara Basseres Novais, Beatriz Rocha Ilidio Rodrigues, Flávia Oliveira Borges Pereira, Alan Gonçalves Amaral, Sofya Castilho Lapa, Lucas Lopes Maldonado, Pedro Víctor-Carvalho, Isabela Aparecida Moretto, Hans Rolando Zamora-Obando, Mariana Conceição da Silva, Ana Paula Samogim, Ingridi Rafaela de Brito, Maria das Graças de Souza Carvalho, Antonio Thiago Pereira Campos, Michelle Bueno de Moura Pereira Antunes, Carlos Lenz Cesar, Hernandes F. Carvalho, Ana Valéria Colnaghi Simionato, André Alexandre de Thomaz, Aline Mara dos Santos

**Affiliations:** 1 Department of Structural and Functional Biology, Institute of Biology, University of Campinas (UNICAMP), Campinas, Brazil; 2 Department of Analytical Chemistry, Institute of Chemistry, University of Campinas (UNICAMP), Campinas, Brazil; 3 Department of Quantum Electronics, Gleb Wataghin Institute of Physics, University of Campinas (UNICAMP), Campinas, Brazil; 4 National Institute of Science and Technology on Photonics Applied to Cell Biology (INFABIC), University of Campinas (UNICAMP), Campinas, Brazil; 5 Universidade Federal do Ceará (UFC), Fortaleza, 60440-900 Ceará Brazil; 6 Department of Basic Life Sciences, Institute of Life Sciences, University of Juiz de Fora (UFJF), Governador Valadares, Brazil; 7 National Institute of Science and Technology in Bioanalytics (INCTBio), Campinas, Brazil

**Keywords:** Cardiomyocytes, Differentiation, Metabolic shift, Mitochondria, ROS, Biochemistry, Cardiology, Cell biology, Physiology

## Abstract

Cardiomyocyte differentiation is a complex process involving significant metabolic remodeling, but its impact on cellular redox state and cell damage remains poorly understood. Using metabolomics, biophysical, and biochemical approaches, we characterized, in vitro, the metabolic shift of differentiating cardiomyocytes and its implications for oxidative damage. We found that differentiating cardiomyocytes undergo a broad metabolic reprogramming from a glycolytic to an oxidative state, marked by increased activity in key pathways, including malate-aspartate shuttle, glutathione metabolism, and tricarboxylic acid cycle. This metabolic transition was associated with mitochondrial enlargement and increased reactive oxygen species (ROS) production. Intriguingly, despite ROS increase, differentiated cells maintained similar levels of DNA damage as cardiomyoblasts and were more resistant to a H₂O₂ challenge. Our findings suggest that metabolic adaptations during cardiomyocyte differentiation enhance their capacity to mitigate oxidative stress damage, providing an adaptive avenue that enables cardiomyocyte survival upon exposure to an oxygen-rich environment.

**Supplementary Information:**

The online version contains supplementary material available at 10.1038/s41598-026-35263-5.

## Introduction

Cardiovascular disease (CVD) is the leading cause of death globally, accounting for approximately 32% of all deaths worldwide. Annually, CVD claims the lives of 19.8 million people, with three-quarters of these deaths occurring in low and middle-income countries (WHO, 2025)^1^. As the adult human heart is unable to replace extended injury areas due to the limited proliferative capacity of differentiated cardiomyocytes, the damaged tissue is mainly replaced by fibrosis, a process contributing to pathological cardiac remodeling and heart failure^2,3^. Therefore, a comprehensive understanding of cardiomyocyte differentiation may provide new perspectives for more effective therapies to reduce cardiac morbidity and mortality.

During differentiation, cardiomyocytes go through a metabolic transition, shifting their primary energy substrate from glucose to fatty acids^4–6^. This metabolic alteration coincides with birth, when differentiating cardiomyocytes are exposed to an oxygen rich environment^4–6^. At this stage, cardiomyoblasts initiate a final round of DNA replication without cytokinesis, resulting in large tetraploid (human) or binucleate (rodent) cardiomyocytes. Afterwards, postmitotic cells undergo hypertrophic growth, characterized by the synthesis of contractile machinery components and mitochondrial proliferation and enlargement^4–6^. Given that mitochondria are the largest source of reactive oxygen species (ROS) production, the increase in mitochondrial mass could culminate in elevated oxidative stress^7^. While ROS are essential for regulating cell signaling, including differentiation^8^, excess levels can trigger DNA damage and activate the DNA damage response (DDR) pathways, ultimately leading to cell cycle arrest and maturation of cardiomyocytes^9–12^. This coordinated programming suggests an intrinsic link between cardiomyocyte metabolism, differentiation, and proliferative capacity. However, although cardiomyocyte differentiation is an active and expanding field of research, the specific metabolic alterations that accompany cardiomyocyte maturation are yet to be fully elucidated.

Using an integrative approach that combined gas chromatography coupled to mass spectrometry (GC-MS) untargeted metabolomics with biophysical and biochemical assays in H9c2 cardiomyocytes, we identified the metabolic shift and key pathways modulated during the transition from proliferative cardiomyoblasts to differentiated cardiomyocytes. Our Fluorescence Lifetime Imaging Microscopy^13–17^ (FLIM) analysis corroborated a metabolic shift in cardiomyocytes from a glycolytic to an oxidative state. We also characterized the specific pathways these metabolites are involved in and how they were modulated by this shift, such as the malate-aspartate shuttle, amino acid metabolism, the TCA cycle (also known as the Krebs cycle), and the metabolism of glutathione, glutamate, and carnitine. Our data showed an enlarged mitochondrial pattern and an increased ROS production coinciding with the terminal stages of differentiation, demonstrating the relationship between mitochondrial function and increased oxidative stress related to differentiation. However, despite this elevated ROS levels, cardiomyocytes maintained a similar amount of DNA strand breaks compared to cardiomyoblasts, as measured by γ-H2AX foci count. This indicates that the cardiomyocyte DNA damage repair machinery is capable of handling the DNA breaks resulting from the increased oxidative stress during differentiation. Moreover, after a H_2_O_2_ challenge, cardiomyocytes were more efficient to repair the damaged DNA, leading to enhanced cell survival. Our findings identify the specific metabolic changes associated with the overall shift to oxidative phosphorylation and suggest an intriguing link between the metabolic remodeling of cardiomyocytes and their enhanced capacity to mitigate the oxidative damage during differentiation. This provides new insights on the adaptive mechanism that enables cardiomyocyte survival upon exposure to a rich oxygen environment.

## Results

### Cardiomyoblasts differentiation resulted in polynucleated cardiomyocytes containing reduced Ki67 expression

**Fig. 1:**
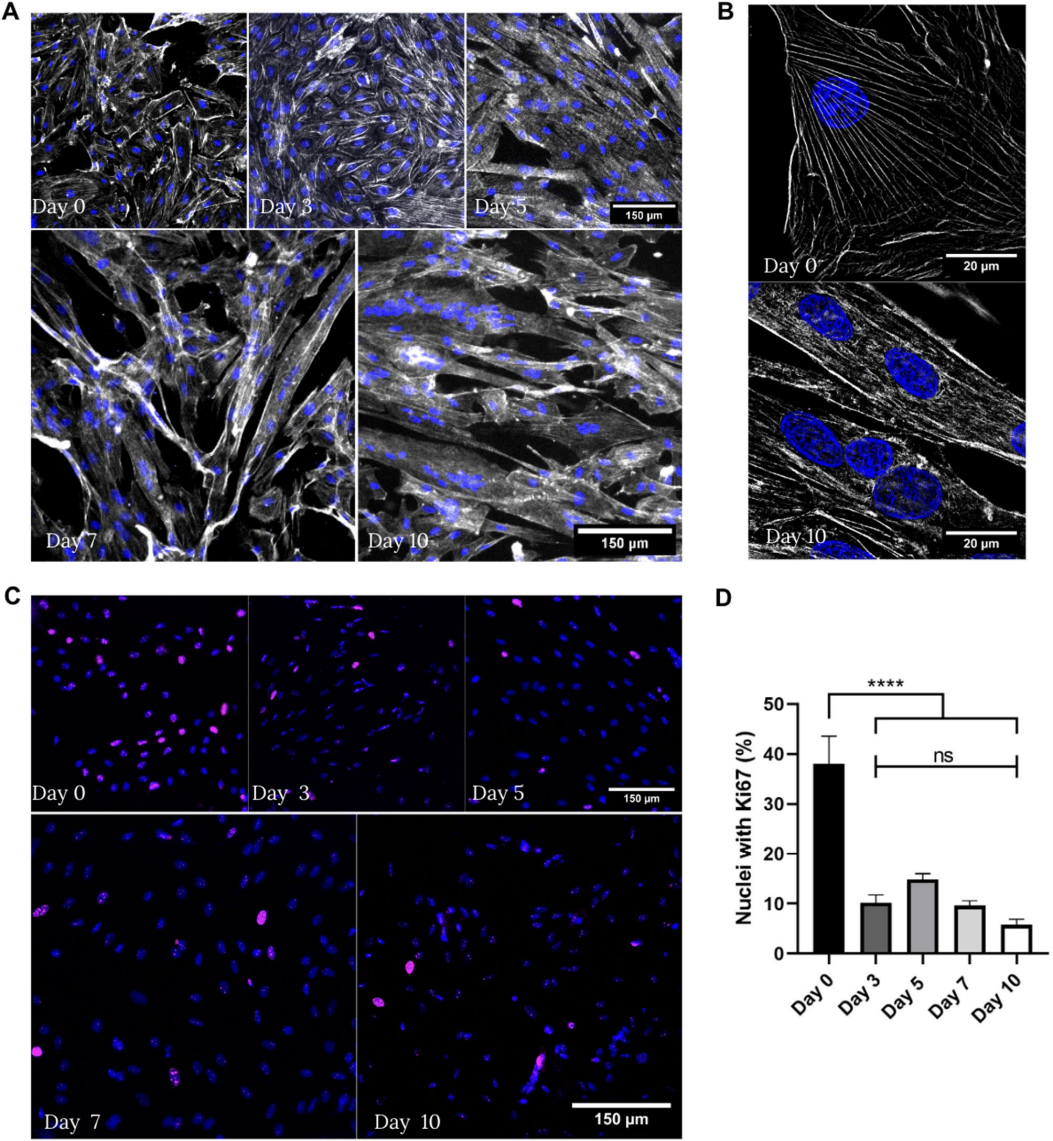
**(A-B)** Immunofluorescence staining of actin (phalloidin in gray) and nuclei (DAPI in blue) showing H9c2 differentiation throughout the indicated days. **(C)** Ki67 staining of differentiating cardiomyocytes, from day 0 (without serum reduction and retinoic acid addition) to day 10 of differentiation, are shown. **(D)** Bar graph shows the percentage of cell nuclei stained with Ki67. Nuclei are considered stained if Ki67 signal covers 30% or more of the nuclei’s total area. Day 0 *n* = 277 cells; Day 3 *n* = 321 cells; Day 5 *n* = 475 cells; Day 7 *n* = 476 cells; Day 10 *n* = 548 cells. Data was represented as mean ± SEM. Normality was assessed using Shapiro-Wilk test and statistical significance was measured using One-way ANOVA test, ****p < 0.0001.

H9c2 cells and their differentiation into cardiomyocytes-like cells had already been well characterized and widely used in many previous studies, including those related to differentiation and cardiovascular research^18–21^. To assert a successful differentiation of H9c2 cardiomyoblasts (CMB) into cardiomyocytes (CM), we used Super Resolution by Structured Illumination Microscopy (SR-SIM). Our images showed the mononucleated pattern and the fusiform morphology of H9c2 CMB (Fig. 1A-B, **Supplem.** Figure 1). During the initial three days of differentiation, cell confluency increased, suggesting a phase of active proliferation. While most cells remained mononucleated, their boundaries became less defined due to this growth (Fig. 1A-B**)**. Importantly, this proliferative phase was transient, as the expression of the proliferation marker Ki67 was significantly reduced after day 3 in the differentiating CM, as observed by its decreased nuclear localization (Fig. 1C-D). Between 7 and 10 days, differentiating cells showed a dramatic change in their morphology, fusing together to generate larger polynucleated cells organized in a fibrous shape-like phenotype with reduced expression of Ki67, as expected for CM.

### Fluorescence lifetime imaging demonstrated the metabolic shift in cardiomyocytes following differentiation

**Fig. 2:**
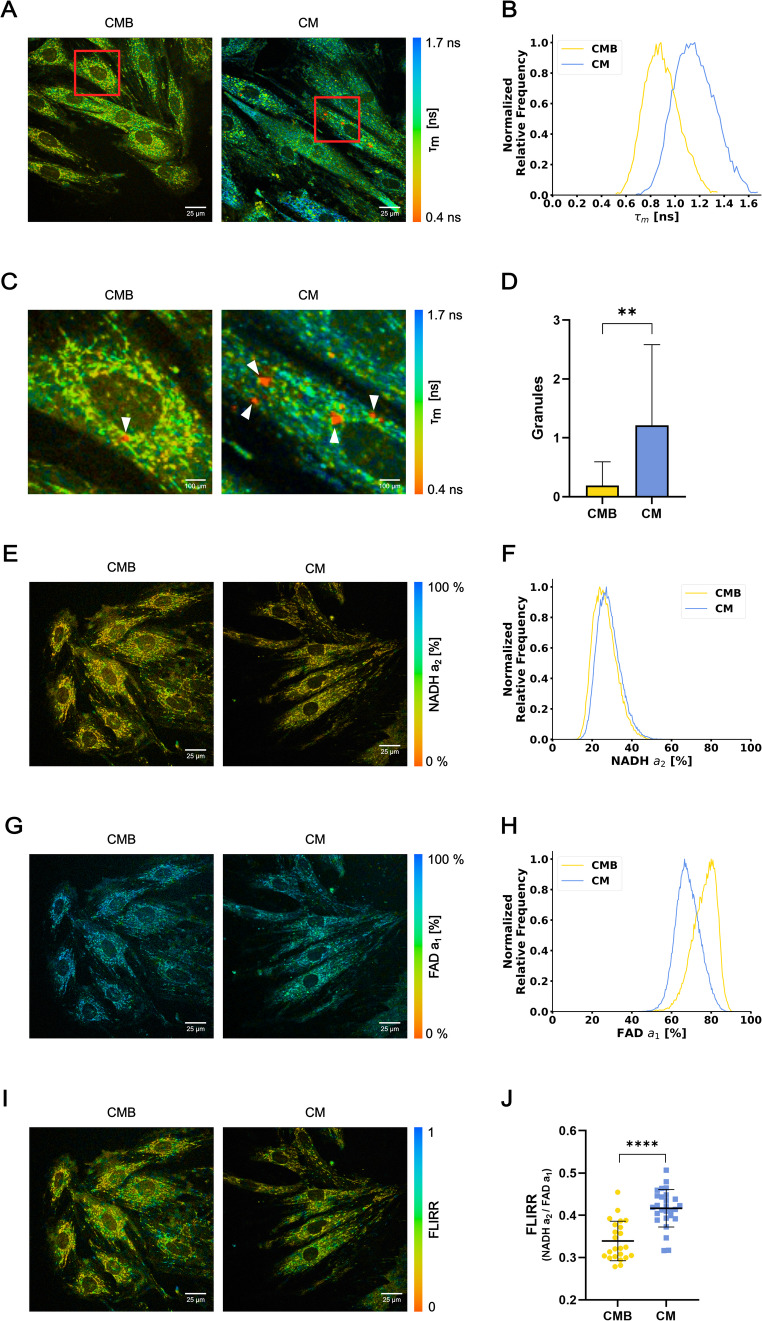
**(A)** FLIM images color coded by the mean fluorescence lifetime. **(B)** Histogram of the mean fluorescence lifetime of CMB and CM, Cohen’s d = 1.75. CMB *n*=21 cells; CM *n*=14 cells. **(C)** Insets of **(A)** showing granules of short fluorescence lifetime (in red). **(D)** Bar graph of the number of granules per cell of CMB and CM. Data are represented by Mean±SD. Mann-Whitney test was used to compare groups (*p*=0.0019). CMB *n*=21 cells; CM *n*=14 cells. **(E)** FLIM images color coded by the a_2_ component of NADH. **(F)** Histogram of the a_2_ component of NADH in CMB and CM, Cohen’s d=0.34. CMB *n*=23 cells; CM *n*=27 cells. **(G)** FLIM images color coded by the a_1_ component of FAD. **(H)** Histogram of the a_1_ component of FAD in CMB and CM, Cohen’s d=1.37. CMB *n*=23 cells; CM *n*=27 cells. **(I)** FLIM images color coded by the FLIRR coefficient. **(J)** Plot of the FLIRR coefficient (NADH*a2*/FAD*a1*) of CMB and CM. Data are represented by Mean±SD. Unpaired t test was used to compare groups (*p* < 0.0001). CMB *n*=23 cells; CM *n*=27 cells.

To evaluate, in vitro, whether differentiation induces a metabolic shift in cardiomyocytes, we employed FLIM in living cells. FLIM enables the identification of different chemical environments of endogenous fluorophores in live cells by the measurement of fluorescence lifetime, giving insight into metabolic alterations. This is possible because fluorescence lifetime of fluorophores changes on function of their chemical environment^17,22^. Endogenous fluorophores enable non-invasive imaging of biological samples with minimal disruption to their natural state. For example, the autofluorescence of metabolic coenzymes *(I)* reduced nicotinamide adenine dinucleotide (NADH) and *(II)* flavin adenine dinucleotide (FAD) makes them label-free probes for visualizing and analyzing cellular metabolism in real-time. First, we evaluated the broad fluorescence lifetime, collecting all fluorescence signals below 690 nm, of live cardiomyoblasts and cardiomyocytes. The images (Fig. 2A-B) show the distribution of different lifetimes of endogenous fluorescent compounds in the cytoplasm. Our data showed that differentiated cells had a longer mean fluorescence lifetime (1.16 ± 0.17 ns - Mean ± SD) than the undifferentiated (0.89 ± 0.14 ns) (Fig. 2A-B), confirming a remodeling of the metabolism during differentiation. Interestingly, an increased number of granules with a shorter mean lifetime was observed in CM (1.21 ± 1.37 granules/cell - Mean ± SD) as compared to CMB (0.19 ± 0.40 granules/cell). The short fluorescence lifetime of these granules indicates peroxidized lipids accumulation in lipofuscin, which serves as an indicator of oxidative stress^23,24^ (Fig. 2C-D). As these granules were too small, their fluorescence lifetime did not significantly alter the mean lifetime distributions. Next, we detected the fluorescence lifetime of NAD(P)H and FAD, using 445 ± 45 nm BP and 535 ± 22 nm BP filters, respectively, allowing the calculation of the Fluorescence-Lifetime Redox Ratio (FLIRR)^25,26^, which serves as a concentration-independent metabolic index that allows distinction between glycolysis and oxidative phosphorylation by quantifying the ratio of bound NAD(P)H to bound FAD within the cell. The coenzymes NAD(P)H and FAD can exist in free or protein-bound states. NAD(P)H presents a short lifetime when free and a long lifetime when bound, whereas FAD exhibits the opposite behavior. Consequently, by measuring fluorescence lifetime, it is possible to quantify the bound and free fractions of these coenzymes.

After fitting a biexponential decay curve to the measured data, we found that differentiated cells presented very similar NAD(P)H a_2_ (28.4 ± 5.8% - Mean ± SD) distribution as undifferentiated cells (26.4 ± 5.8%), but noticeably different FAD a_1_ distribution (68.2 ± 6.0% against 76.4 ± 6.0%) (Fig. 2E-H). With these parameters, mean FLIRR was calculated for each cell, showing that CM cells had higher FLIRR (0.42 ± 0.04 - Mean ± SD) than CMB cells (0.34 ± 0.05) (Fig. 2I-J). A higher FLIRR in CM suggests a shift from glycolysis in CMB to oxidative phosphorylation in CM. This finding corroborates, in vitro, the metabolic transition expected in mammalian cardiomyocytes at the late stage of differentiation^5^.

### GC-MS analysis elucidates the metabolic shift in cardiomyocytes undergoing differentiation

**Fig. 3:**
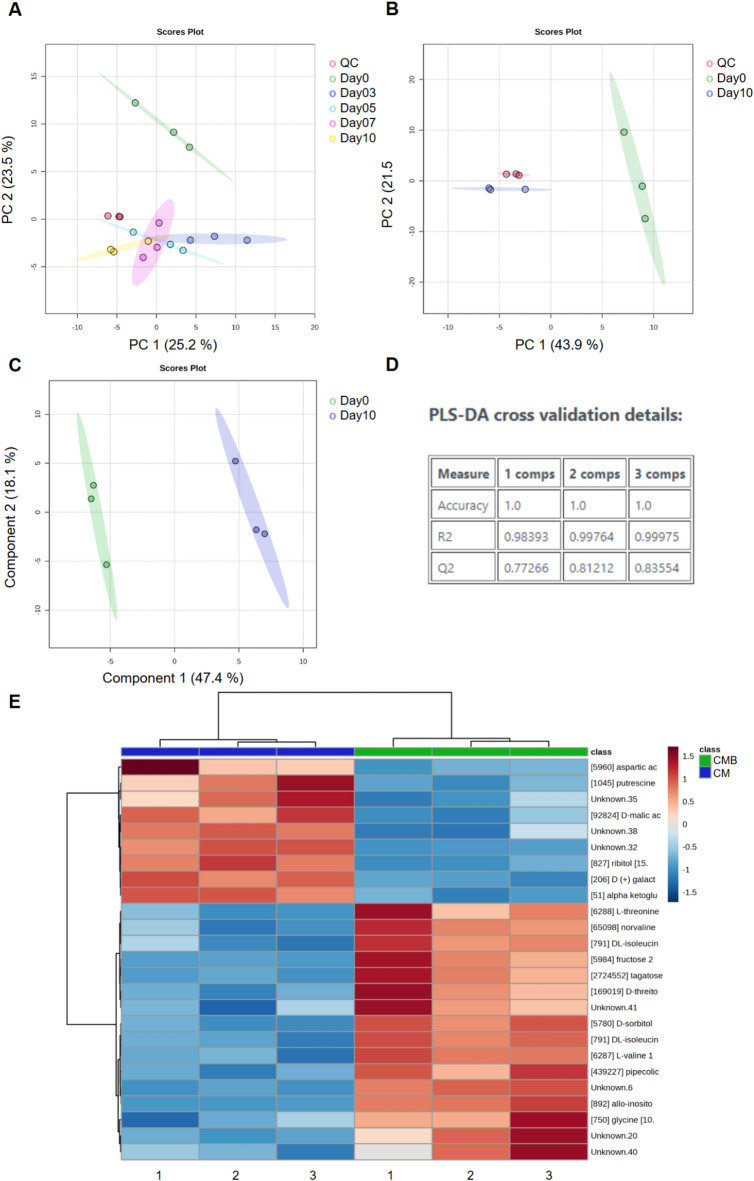
**(A)** PCA score plot for samples: Day 0 (green), Day 3 (dark blue), Day 5 (light blue), Day 7 (pink), Day 10 (yellow), and QCs (red). **(B)** PCA score plot for day 0 (green), day 10 (blue) and QCs (red). **(C-D)** PLS-DA plot, Day 0 (green) and Day 10 (blue) and Cross-validation results for the indicated components. **(E)** Heat map representing significantly different (*p* < 0.05) known and unknown metabolites between undifferentiated (Day 0) and differentiated (Day 10) cardiomyocytes. Group identity (Day 0 or Day 10, biological replicates *n* = 3) is indicated at the top of the figure, while individual samples are indicated at the bottom. Data were normalized, log-transformed, auto-scaled and compared via Student’s t-test. CMB: Cardiomyoblasts; CM: Cardiomyocytes.

To identify the metabolites altered during differentiation, we employed the GC-MS technique to analyze the endometabolome content of cardiomyoblasts (day 0) and differentiating cardiomyocytes (days 3, 5, 7, and 10). Data were processed by MS-Dial software, version 4.80, resulting in 109 molecular features. Principal Component Analysis (PCA) was performed to observe grouping trends between samples and to assess the absence of instrumental variation, i.e. the grouping of the Quality Control samples (QCs). Results showed a grouping pattern between the samples and clustered QCs (Fig. 3A), which guarantees that any variation observed within the groups of samples was related to biological sources rather than sample preparation or analyses. Among the 109 molecular features, principal component PC2 clearly distinguished Day 0 from Days 3, 5, 7, and 10 of differentiating cardiomyocytes. Our results confirmed that the transition from cardiomyoblasts to cardiomyocytes is accompanied by a significant shift in global metabolite profiles (Fig. 3A). Since this study aimed to characterize the metabolic shift that marks the transition to CM, we performed the following analysis comparing Day 0 (CMB) and Day 10 (CM) of differentiation. As expected, the principal component PC1 clearly distinguished the groups, confirming the shift in global metabolites profile (Fig. 3B).

To identify metabolites with the most significant contributions to the differences between the groups, we performed a Partial Least Squares-Discriminant Analysis (PLS-DA) plot and Variable Importance in Projection (VIP) scores (Fig. 3C-D). PLS-DA models and VIP scores were built without the QC samples to maximize the discrimination between groups since it is a supervised method. Cross-validation results indicated that the models were well-adjusted (R²_endo_ > 0.999) and effective at predicting the behavior of the samples (Q² > 0.835). Using the parameters established for defining the significant features, a total of 24 metabolites displayed altered levels between CMB (Day 0) and CM (Day 10) (Fig. 3E; Table 1). The heat map of the known and unknown metabolites revealed distinct clustering between the groups. In addition to the metabolic shift identification, these results demonstrate the potential of metabolomics to accurately classify undifferentiated and differentiated cardiomyocytes.

**Table 1:** Known metabolites up and down regulated during cardiomyocyte differentiation identified by GC-MS.

t_*r*_ (min)	Metabolite	*p* Value	FDR	VIP Score	Fold Change	CM x CMB
7.296	L-Valine	0.00069	0.00717	1.437	-0.940	↓
9.468	Norvaline	0.00300	0.02155	1.402	-1.849	↓↓
9.868	Pipecolic Acid	0.00228	0.02061	1.411	-1.039	↓↓
10.224	L-Threonine	0.00731	0.03938	1.365	-1.393	↓↓
10.225	L-Isoleucine	0.00473	0.02967	1.385	-2.325	↓↓↓
10.456	Glycine	0.01439	0.06440	1.322	-1.487	↓↓
10.735	Glyceric acid	0.04012	0.13468	1.221	0.664	↑
12.002	Aspartic acid	0.03012	0.11324	1.255	1.375	↑↑
12.794	Malic acid	0.00241	0.02061	1.409	0.960	↑
12.954	Threitol	0.00754	0.03938	1.363	-1.144	↓↓
13.338	Glutamic acid	0.04006	0.13468	1.221	1.872	↑↑
13.859	Oxoglutaric acid	0.00026	0.00346	1.449	0.992	↑
15.660	Ribitol / Xylitol / Arabitol	0.00029	0.00346	1.448	1.347	↑↑
15.709	Dopamine / Citric Acid	0.00927	0.04588	1.351	2.049	↑↑↑
17.011	Tagatose / Mannose / Glucose	0.00321	0.02155	1.400	-3.163	↓↓↓↓
17.245	Inositol	0.00007	0.00249	1.458	-3.645	↓↓↓↓
17.288	Fructose	0.00306	0.02155	1.401	-3.154	↓↓↓↓
17.662	Galactose	0.00012	0.00281	1.455	3.472	↑↑↑↑
17.898	Sorbitol	0.00026	0.00346	1.449	-1.906	↓↓

Our metabolomics approach revealed 19 known metabolites significantly altered after cardiomyocyte differentiation (Table 1; **Supplem.** Table 1; **Supplem.** Figs. 2 and 3). Glycolysis intermediates (glucose and fructose) were significantly lower in differentiated cardiomyocytes while concentrations of metabolites involved in TCA cycle (citric acid, malate, and oxoglutarate) and malate-aspartate metabolism (aspartate, glutarate, and oxoglutarate) were higher. Interestingly, a set of amino acids were significantly lower in the Day 10 group, including glycine, isoleucine, threonine, and valine, while aspartic acid and glutamic acid, metabolites of the aspartate-malate shuttle, were higher. Inositol, which is metabolized to phosphatidylinositol and converted to phosphatidylinositol - 4,5 - bisphosphate, was decreased in cardiomyocytes. On the other hand, dopamine, an unstable metabolite, whose catabolism results in the production of reactive oxygen species (ROS), was upregulated.

### MetaboAnalyst tools revealed the most enriched pathways modulated during cardiomyocyte differentiation

**Fig. 4:**
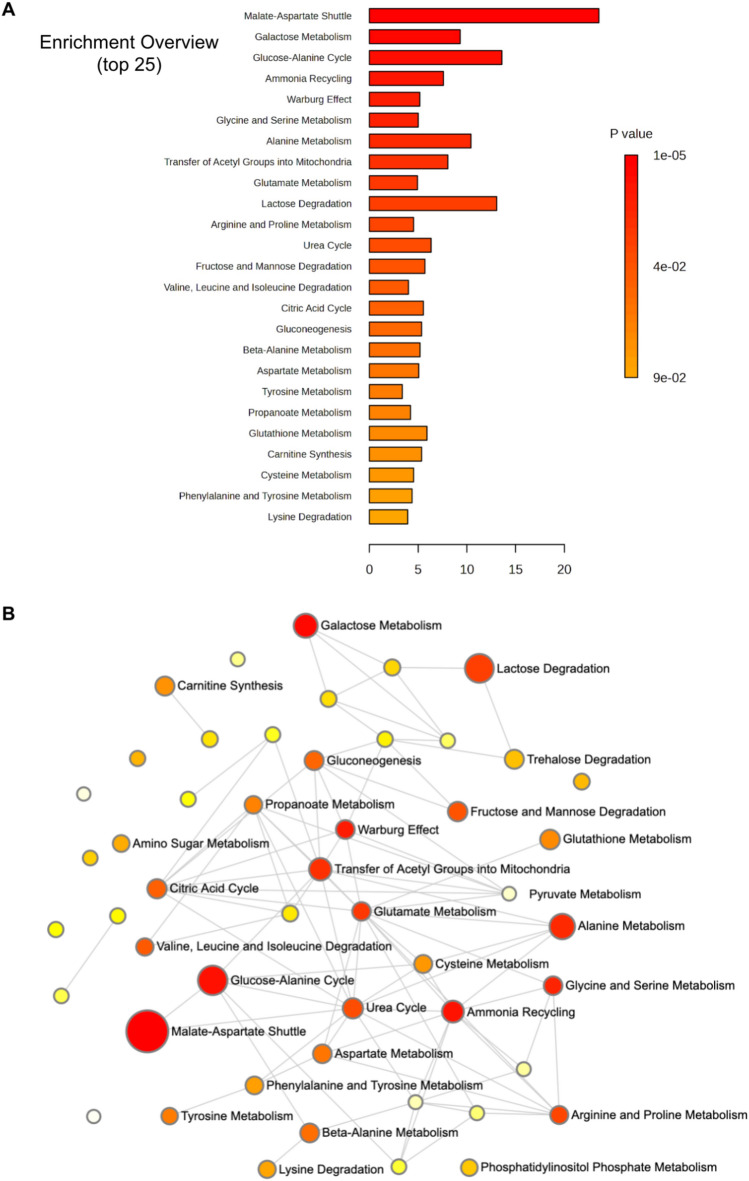
Overview of enriched metabolic pathways based on metabolomics analysis. **(A)** The bar plot shows the top 25 pathways ranked by enrichment ratio, with bar colors indicating the statistical significance (p-value). **(B)** The network plot illustrates the interconnections among pathways, where node size reflects enrichment ratio and node color corresponds to the p-value.

In order to associate the modulated metabolites to the corresponding metabolic pathways, we used the Enrichment Analysis tool of MetaboAnalyst 5.0. Twenty-five pathways were identified as enriched pathways presenting the most significant alteration between CMB and CM in the metabolome (Fig. 4). This method employs over-representation analysis (ORA) to identify metabolic pathways or metabolite sets that exhibit statistically significant enrichment for the molecules of interest^27^. Figure 4 presents a graph and network depicting the metabolic pathways modulated during cardiomyocyte differentiation.

**Fig. 5:**
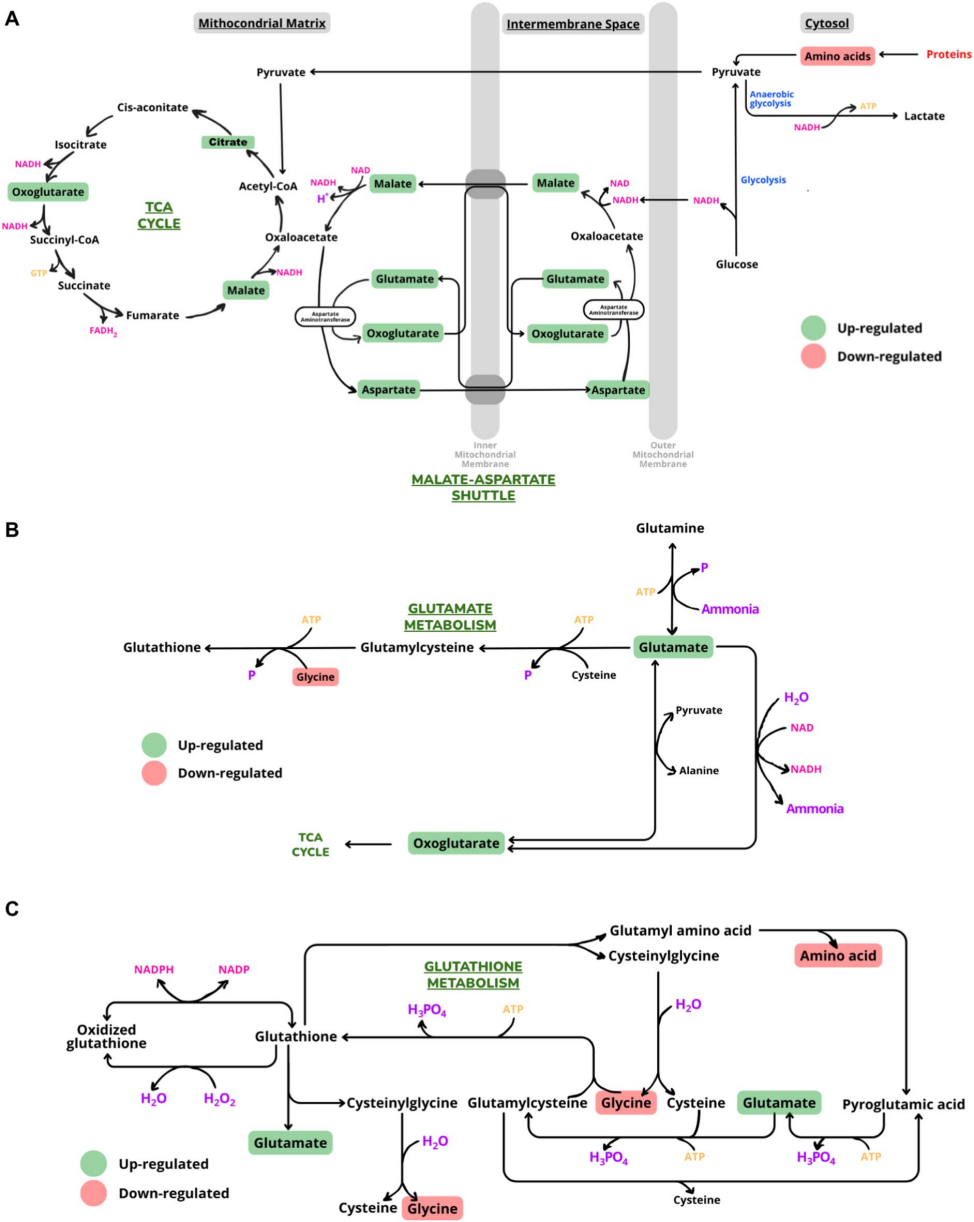
Schematic representations of TCA cycle (Krebs cycle). **A**. Malate-aspartate shuttle. Malate is transported into the mitochondrial matrix via malate-α-ketoglutarate transporter, where it is converted to oxaloacetate in the TCA cycle by the generation of NADH. Oxaloacetate is converted to aspartate and transported to intermembrane space by the aspartate-glutamate transporter, where it is converted to oxaloacetate. Therefore, the NADH produced by glycolysis in the cytosol transfers its reducing equivalents to oxaloacetate in the intermembrane space to produce malate. **B**. Glutamate metabolism. Glutamate is transported from the cytosol to the mitochondrial matrix, where it undergoes oxidative deamination to produce α-ketoglutarate and ammonia (NH_4_^+^), generating NADH. Excess ammonia can combine with glutamate to produce glutamine, which can participate in the urea cycle or be used in biosynthetic pathways. Glutamate also can bind to cysteine and glycine, a process that consumes ATP and generates glutathione. **C**. Glutathione metabolism. Glutathione is composed of three amino acids: glycine, glutamate, and cysteine. Glutathione participates in oxidation-reduction reactions, donating electrons to H₂O₂ to form H₂O and producing glutathione disulfide, its oxidized form. This reaction is catalyzed by glutathione peroxidase. Glutathione reductase participates in the reaction in which electrons from NADPH are used to reduce glutathione disulfide regenerating glutathione.

To visualize the enriched metabolic pathways, we employed the Small Molecule Pathway Database (SMPDB) and KEGG platforms^28–30^. Our results revealed alterations in metabolites related to amino acids pathways including the metabolism of (*I*) alanine, (*II*) glycine and serine, (*III*) arginine and proline, (*IV*) aspartate, and (*V*) valine, leucine, and isoleucine degradation. These amino acid pathways are related to protein degradation for amino acids transformation into energy substrates for the TCA cycle. Moreover, the variation of oxoglutarate levels with the increased malate, aspartate, and glutamate concentrations indicate a modulation of the malate-aspartate shuttle pathway, which provides oxaloacetate to the TCA cycle and acts on the transfer of electrons from glycolysis to the mitochondrial matrix, through the regeneration of NADH (Fig. 5A). Although we found the Warburg effect was also enriched in our analysis, specific metabolites driving this enrichment were almost exclusively upregulated TCA cycle intermediates (**Supplementary** Table 1). We interpret this observation not as a true Warburg effect, but rather as an indication of the suppression of glycolysis (the primary Warburg pathway) in favor of the enhanced activity of the TCA cycle. The modulation of the glutamate metabolism is also linked with the regeneration of a TCA cycle intermediate, oxoglutarate (Fig. 5B), indicating the positive modulation of this cycle at the late stage of cell differentiation. The glutathione metabolism was also modulated during cardiomyocyte maturation. After differentiation, cardiomyocytes must be able to cope with an increased use of oxygen in energy metabolism which may result in oxidative damage. To decrease the damage generated by the increased ROS production, antioxidant mechanisms, including the glutathione metabolism, may be employed to neutralize the effects of ROS in the cellular environment^31^. Glutathione is oxidized by reactive oxygen species and is easily reconstituted in the presence of the amino acids cysteine, glycine, and glutamate (Fig. 5C). The modulation of glycine and glutamate metabolites concentrations indicated a regulation of the glutathione cycle in the late stage of cardiomyocyte differentiation^32^. In addition, urea cycle, ammonia recycling, and Carnitine Synthesis were detected among the metabolic pathways modulated during cardiomyocyte differentiation.

### Differentiated cardiomyocytes exhibit higher ROS levels but enhanced capacity to mitigate the effects of oxidative stress

**Fig. 6:**
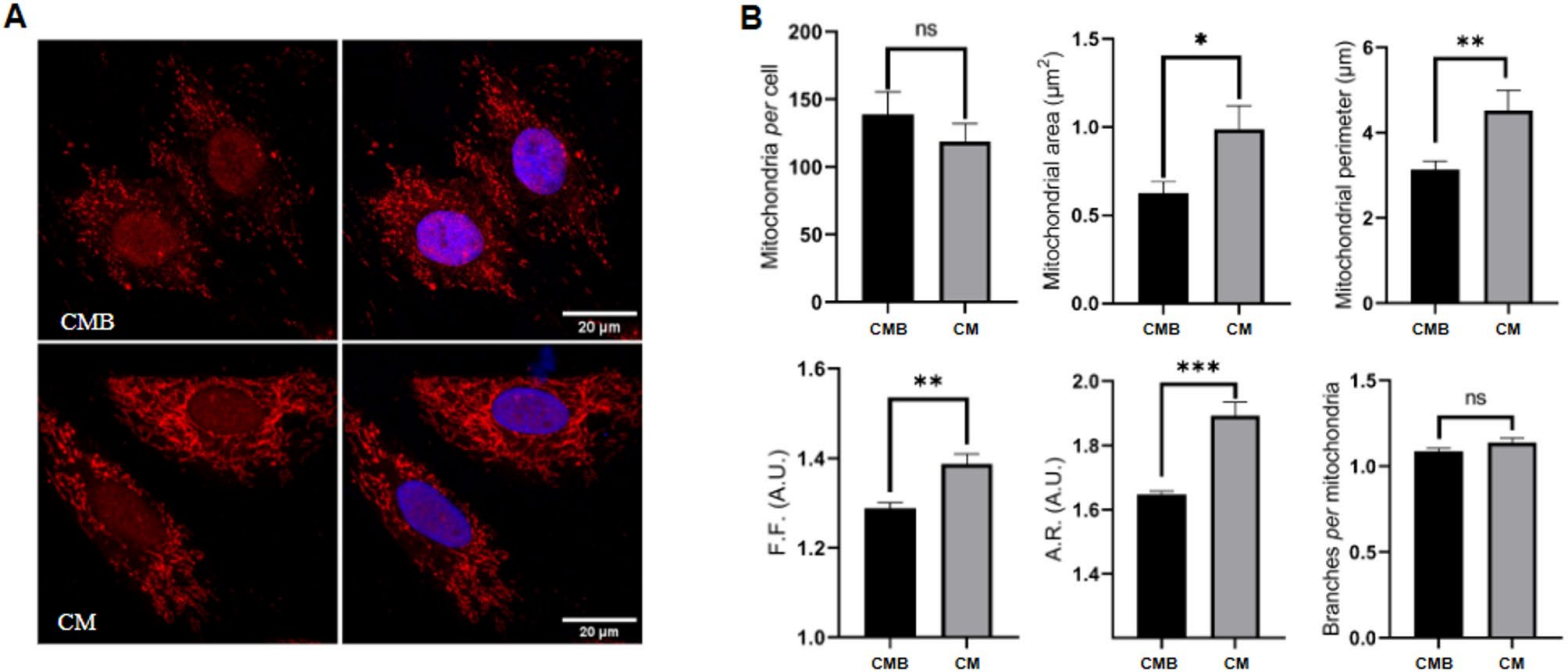
Cardiomyocytes presented larger and more elliptical mitochondria than cardiomyoblasts. (**A**) Immunofluorescence staining of mitochondria (MitoTracker™ DeepRed in red) and nuclei (DAPI in blue) showing H9c2 differentiation in days 0 (CMB) and 10 (CM). (**B**) Quantitative analysis and comparison of mitochondria performed on H9c2 cells under differentiation in CMB (*n* = 12 cells) and CM (*n* = 12 cells). Data are represented by means ± SEM. Kolmogorov-Smirnov test was used to assess normality and t test or Mann-Whitney test were used to compare the groups; *p ≤ 0.05; ***p < 0.001. AR, aspect ratio; FF, form factor; A.U., arbitrary units.

It has been proposed by Garber and Lee (2021), that mitochondrial maturation and subsequent shifts in redox homeostasis may be a driver of cardiomyocyte differentiation in the postnatal period, when cells are exposed to a rich oxygen environment^10,33^. However, the characterization of the redox balance effects during cardiomyocyte maturation remains poorly understood. First, we investigated whether the H9c2 cardiomyocyte differentiation impacts mitochondrial morphology and the redox balance. Our mito-tracker analysis showed a notable increase in mitochondrial area (*p* < 0.01) in CM (Fig. 6A-B). However, no differences in the number of mitochondria between CMB and CM was observed in the H9c2 model (Fig. 6B). Morphologically, mitochondria on CM exhibit a more elliptical shape compared to those on CMB, as both the F.F. (form factor) and A.R. (aspect ratio) of CM samples were increased (Fig. 6B). Our data demonstrated that, despite the overall number of mitochondria being unchanged, the mitochondria of CM were larger and less round, resulting in increased area (Fig. 6B). No alterations were detected in the network profile when assessing mitochondria branch pattern (Fig. 6B).

**Fig. 7:**
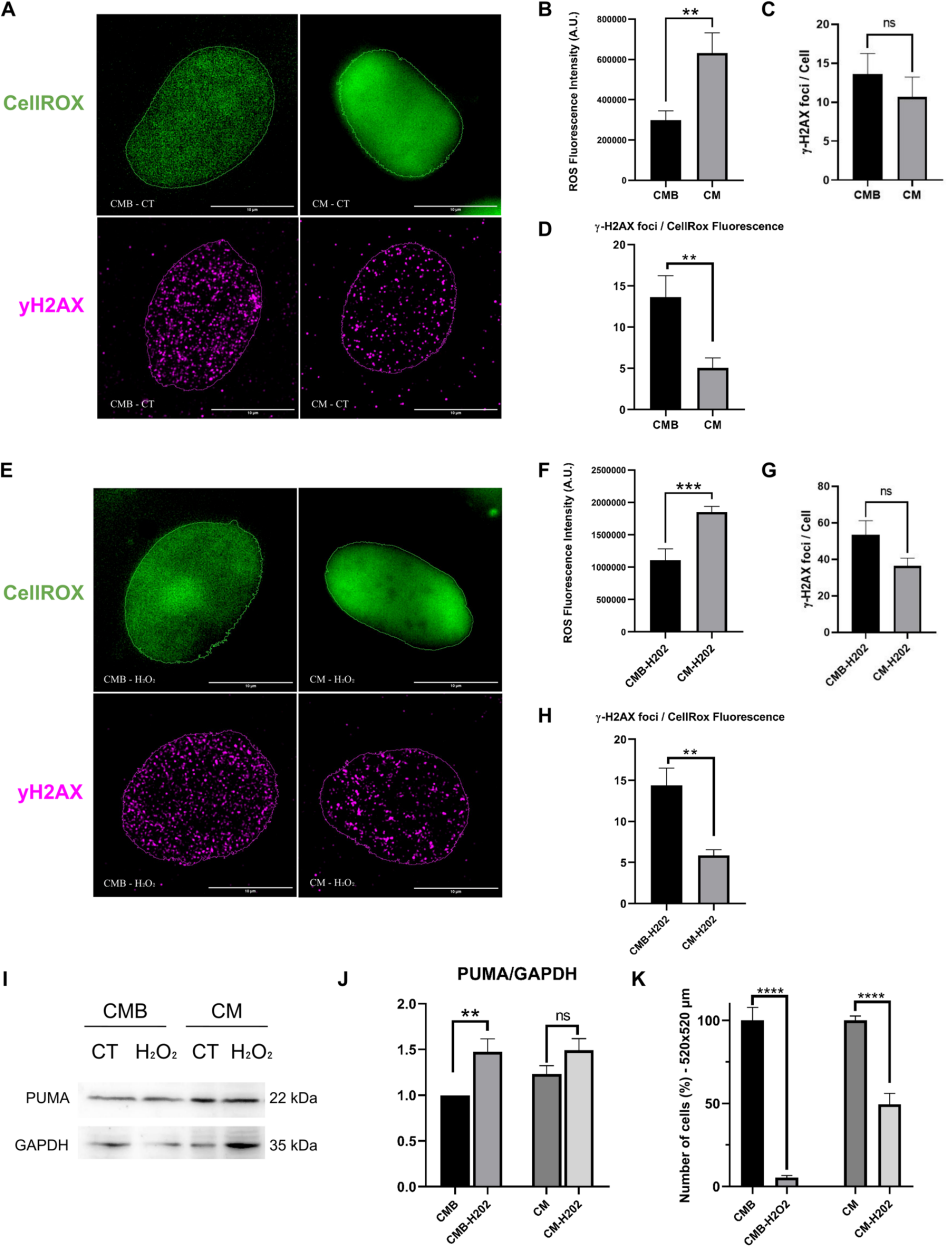
Cardiomyocytes presented enhanced capacity to mitigate the effects of oxidative stress. **(A)** Images of CMB and CM showing, on the top panels, ROS detection using CellRox fluorescent staining (Widefield resolution), and on the bottom panels γ-H2AX foci (Super-resolution). **(B)** Quantitative analysis and comparison of Mean Fluorescence Intensity (MFI) of CellROX measured on CMB (*n* = 23 cells) and CM (*n* = 19 cells). **(C**) Quantitative analysis of γ-H2AX foci measured on CMB (*n* = 16 cells) and CM (*n* = 16 cells), as indicated. **(D)** Graph indicating the proportion of γ-H2AX foci per cell relative to the mean ROS measurement of each group. (**E)** Images of CMB and CM treated with H_2_O_2_ showing, on the top panels, ROS detection using CellRox fluorescent staining (Widefield resolution), and on the bottom panels γ-H2AX foci (Super-resolution). **(F)** Quantitative analysis and comparison of MFI of CellROX measured on CMB (*n* = 9 cells) and CM (*n* = 17 cells) treated with H_2_O_2_. **(G)** Quantitative analysis of γ-H2AX foci measured on CMB (*n* = 39 cells) and CM (*n* = 25 cells) treated with H_2_O_2_. **(H)** Graph indicating the proportion of γ-H2AX foci per cell relative to the mean ROS measurement of each group. **(I)** Immunoblot of PUMA. GAPDH was used as loading control. **(J)** Bar graph of PUMA. *n* = 6 biological replicates. **(K)** Bar graph shows the percentage of cell survival after H_2_O_2_ treatment, as indicated. All data were represented as means ± SEM. Normality was assessed using Kolmogorov-Smirnov test, and t test or Mann-Whitney test were used to compare the groups; *p ≤ 0.05; ****p < 0.0001. CMB: Cardiomyoblasts; CM: Cardiomyocytes. A.U.: arbitrary units.

Next, as metabolomics analysis indicated a modulation of the glutathione metabolism and the mito-tracker analysis showed increased mitochondria area after differentiation, we verified the levels of reactive oxygen species (ROS) on H9c2 cells, before and after the differentiation, using the nuclear CellROX™ Green Reagent probe (Fig. 7A). Our data showed that CM exhibited a higher ROS level compared to CMB (Fig. 7B), corroborating the increased number of lipofuscin granules detected by FLIM. Together, the CellROX results and the increased number of lipofuscin granules confirmed an increased oxidative stress in differentiated cardiomyocytes. To investigate the presence of DNA damage associated with increased ROS in differentiated cardiomyocytes, we performed labeling and quantification of γ-H2AX foci, a marker of DNA strand breaks, on CMB and CM under control conditions. The analysis showed that both undifferentiated and differentiated cells exhibited low levels of DNA damage, measured through γ-H2AX foci count (Fig. 7A and C). However, when considering the differences in ROS levels between both cell groups we found that CM cells presented proportionally less DNA breaks than CMB cells (Fig. 7D). These data suggest that the increased mitochondrial area in differentiated cells leads to the higher ROS levels. However, differentiated cardiomyocytes presented no increase in DNA strand breaks, suggesting that its DNA damage repair machinery is more efficient at dealing with oxidative stress than their undifferentiated counterparts.

Next, to investigate the ability of undifferentiated and differentiated cardiomyocytes to cope with high levels of oxidative stress, we challenged cells with hydrogen peroxide (H_2_O_2_) exposure. As expected, H_2_O_2_ treatment further increased ROS levels in both CMB and CM (**Supplem.** Figure 4A). Notably, CM retained higher ROS levels after H₂O₂ exposure, indicating a persistent oxidative stress state in differentiated cells (Fig. 7E and F). This observation aligns with previous reports^18,34^ demonstrating increased ROS in differentiated cardiomyocytes and further suggests that differentiation leads to a disruption in the standard balance between ROS generation and antioxidant defense. We then proceeded to analyse DNA damage during H_2_O_2_ induced oxidative stress. As expected, our results demonstrated an increase in the number of γ-H2AX foci in cells exposed to H_2_O_2_ as compared to the control cells (**Supplem.** Figure 4B). No differences in the overall number of γ-H2AX foci were observed between CMB and CM (Fig. 7G). However, as demonstrated in control conditions, when the difference in ROS levels between CMB and CM treated with H_2_O_2_ was taken into account, we found that CM had proportionally less DNA breaks than CMB (Fig. 7H).

Subsequent experiments revealed that hydrogen peroxide treatment increased PUMA expression in CMB cells, while PUMA expression in CM cells remained unchanged (Fig. 7I and J; **Supplem.** Fig. 5). Finally, CM cells had a higher percentage of survival (~ 50%) after H₂O₂ treatment, whereas CMB cells presented a lower percentage of survival (~ 10%). (Fig. 7K). This data demonstrated that CM were more resistant to oxygen peroxide induced oxidative stress, perhaps due to an enhanced ability to cope with DNA damage caused by ROS, thereby avoiding apoptosis.

## Discussion

Heart maturation requires cardiomyoblasts to differentiate into cardiomyocytes, a process that entails a striking alteration in their structure, function, and energy metabolism. Although some aspects of cardiomyocyte differentiation have been explored, the metabolic shift is poorly understood. In this work we present new information on the metabolic alteration during cardiomyocyte maturation, under in vitro controlled conditions, using metabolomics strategies combined with biophysical and biochemical assays. We observed significant changes in key metabolic pathways during cardiomyocyte maturation. Specifically, we found modulations in the TCA cycle, malate-aspartate shuttle, glutamate, and glutathione metabolism. Concurrently, the metabolism of glycine, serine, alanine, carnitine and inositol were regulated during differentiation. These metabolic shifts coincided with an increase in mitochondrial size and ROS production. Moreover, our biochemical and microscopy assays revealed that these cardiomyocytes had an enhanced capacity to cope with oxidative damage, indicating an adaptive mechanism for promoting cardiomyocyte survival in an oxygen-rich environment.

Our Fluorescence Lifetime Imaging Microscopy (FLIM) data showed a shift of the mean lifetime of broad autofluorescence metabolites in cardiomyocytes towards longer lifetimes (Fig. 2), an indicator of metabolic changes during differentiation. Fluorescence lifetime is dependent on the chemical environment, thus its shift is explained by changes in parameters such as pH, viscosity and/or redox states. FLIRR calculation, with NAD(P)H and FAD fluorescence lifetime data, showed that cardiomyocyte metabolism shifts from predominantly glycolytic to predominantly oxidative after differentiation (Fig. 2J). Metabolic profiling by measuring the intrinsic fluorescence of endogenous metabolites has become a widely used strategy for analyzing live cells, including those undergoing differentiation^35,36^. Additionally, our FLIM data showed an increase in the number of short lifetime granules associated with lipofuscin-containing oxidized lipids in differentiated cells. As it has been previously demonstrated, oxidized lipid associated fluorescence has been reported as oxidative stress markers^23,37^, which corroborates our data on increased ROS production on the late stage of cardiomyocyte differentiation (Fig. 7).

Next, after measuring depletion of several amino acids in differentiated cells (Table 1) we concluded that CM present increased amino acid metabolism. Previous analyses have shown that an increase in amino acid metabolism can be necessary to generate TCA cycle intermediates, an important factor for energy production in cardiomyocytes, thus avoiding intense lactate production through pyruvate transformation^38^. Increased lactate synthesis would compromise energy production and cardiomyocyte function as these cells require large amounts of ATP to maintain the contractile function^4^. This rationale is aligned to our data, as the amino acids valine, threonine, isoleucine, and glycine were decreased after cardiomyocyte differentiation, perhaps due to an increased consumption (Figs. 4 and 5). These amino acids contribute to energy production by providing metabolic intermediates like pyruvate, acetyl-CoA, and succinyl-CoA to fuel the TCA cycle and oxidative phosphorylation^39,40^.

Furthermore, our enriched pathways analysis also showed a modulation of the malate-aspartate shuttle pathway (Figs. 4 and 5). In cardiomyocytes, cytosolic NADH is formed by glycolysis and lactate oxidation. To maximize ATP synthesis, NADH needs to be transported into the mitochondrial matrix to fuel the electron transport chain. Concurrently, the regeneration of cytosolic NAD + is crucial to sustain glycolytic flux and the conversion of lactate back to pyruvate^41^. In the absence of a functional shuttle, glycolysis is maintained by the reduction of pyruvate to lactate, a process that also regenerates NAD+. Consequently, the proper function of the malate-aspartate shuttle leads to a reduction in lactate production, preventing its accumulation in the cell^42–45^. In addition to being an energy substrate, lactate is an important signaling molecule for physiological and pathological events^45,46^. The intracellular accumulation of lactate stimulates lactate dehydrogenase (LDH) towards its oxidation to pyruvate, activating pyruvate dehydrogenase (PDH), which converts pyruvate into acetyl-CoA that enters the TCA cycle for energy production^47^. On the other hand, lactate, through conversion to pyruvate, functions as a redox buffer, which contributes to the oxidation state and can influence the NAD + to NADH ratio within cells^48–50^. When the NAD(P) + to NAD(P)H ratio increases, cells enter an oxidized state and, consequently, undergo accelerated aging in adult hearts, contributing to the development of cardiovascular diseases^50–58^. From our FLIM measurement we can extract information about the ratio of bound/unbound NAD(P)H and FAD. With a double exponential fit, $\:F\left(t\right)={a}_{1}{e}^{-t/{\tau\:}_{1}}+{a}_{2}{e}^{-t/{\tau\:}_{2}}$, to the fluorescence decay curve we obtain two coefficients, a_1_ and a_2,_ for each coenzyme. For NAD(P)H a_1_ is proportional to unbound NAD(P)H and a_2_ is proportional to bound NAD(P)H. For FAD a_1_ is proportional to the bound FAD and a_2_ is proportional to the unbound FAD. Our FLIM data indicates that bound/unbound NAD(P)H ratios do not change significantly in cardiomyocytes, with a tendency toward increased bound NAD(P)H. A more pronounced change is observed in the bound/unbound FAD ratios, with a predominance of unbound FAD (Fig. 2). These findings are consistent with the fact that oxidative phosphorylation consumes NADH, while producing FAD^26^.

Additionally, our data also demonstrated that specific metabolites of glutamate metabolism are pivotal for distinguishing CM from CMB (Figs. 4 and 5**)**. Our finding is interesting, as glutamate metabolism can work to replenish TCA cycle intermediates through oxoglutarate production, thereby supporting oxidative phosphorylation^59^. During anaplerosis, cytosolic glutamine, the glutamate precursor, must be transported through the inner mitochondrial membrane, via a mitochondrial glutamine transporter, and is converted into glutamate, by the enzyme glutaminase (GLS). Subsequently, glutamate is converted into oxoglutarate, by glutamate dehydrogenase 1 (GDH1) or by mitochondrial aminotransferases, which can participate in the TCA cycle, supporting oxidative phosphorylation^60–64^. Oxoglutarate can also be exported from mitochondria to the cytosol to participate in fatty acid biosynthesis and NADH generation^59^. Our findings, demonstrating a significant regulation in glutamate metabolism (Table 1) following the differentiation process, corroborate existing literature. This suggests that the metabolism of glutamate contributes to mitochondrial energy production during differentiation.

Our study also identified glutathione metabolism as a modulated pathway during cardiomyocyte maturation (Figs. 4 and 5). Glutathione (GSH) is an essential part of one of the most important endogenous antioxidant systems of the cell and helps to prevent oxidative damage and cell death^32,65^. Glutathione is formed through two main pathways: either it is synthesized through an ATP, cysteine and glycine-dependent reaction that uses glutamate as a substrate, or it is regenerated from oxidized glutathione by the enzyme glutathione reductase^65^. Interestingly, among the glutathione pathway metabolites, we found that cardiomyocytes have increased glutamate (+ 1.872 fold change) and decreased glycine (-1.487 fold change) levels (Table 1**)**. Although the most rate-limiting step of glutathione metabolism is the availability of cysteine, glycine disponibility also affects GSH synthesis to some extent^32,66^. Since we found an increase in ROS production and an accumulation of lipofuscin granules, we suggest that glutathione metabolism may not be sufficient to counteract ROS production in the final stages of cardiomyocyte differentiation. These findings are in accordance with Chen et al.’s suggestion that antioxidant systems are less active in the heart when compared to other tissues^67^.

As pointed out by Garber and Lee (2021) mitochondrial dynamics and functions are sensitive to metabolic signaling^33^. Our findings on metabolites associated with oxidative phosphorylation and antioxidant defenses, prompted us to investigate how differentiation in this model could affect mitochondria and, by extension, the cell’s redox balance. We found interesting changes in mitochondrial size and shape, namely that differentiated cardiomyocytes had significantly larger and less round mitochondria than undifferentiated cells (Fig. 6). In Birket et al.’s work, increased mitochondria have been linked to factors like transcription co-activator PGC-1 and an enhanced energy-generating capacity^68^. Additionally, Wang et al. has suggested that mitochondrial elongation is correlated to resistance to ROS-induced depolarization, which helps to protect this organelle from mitophagy^69^. Overall, enhanced mitochondrial activity has been found to increase ROS production during certain stages of cardiomyocyte development, as our own data corroborates, so mitochondrial elongation may be a cellular strategy to protect this organelle from excessive damage^33,70^. Furthermore, increased ROS in cardiomyocytes may also be directly related to the differentiation process itself, as it has been demonstrated that oxidative stress plays a complex role in regulating differentiation^71,72^. For instance, while moderate levels of ROS can activate Gata4, promoting cardiomyocyte differentiation, excessive ROS can target Gata4 for proteasomal degradation, inhibiting differentiation^72^. Our results highlight the delicate balance of oxidative stress and corresponding cellular adaptations intrinsic to cardiomyocyte differentiation, emphasizing the importance of precise ROS regulation for optimal cardiac development.

Finally, other studies have found that late stages of cardiomyocyte differentiation are characterized by mitochondrial maturation coinciding with increased ROS production and, consequently, with increased DNA breaks and activation of DNA damage response (DDR) signaling pathways^10,73^. DDR activates a kinase cascade that culminates in the phosphorylation of histone H2AX. Phosphorylated H2AX (γ-H2AX) forms foci at the sites of DNA damage, which serve to recruit the DNA repair machinery to repair the damage. In addition to promoting DNA repair, DDR signaling pathways can also promote cell cycle arrest and loss of cardiomyocyte regenerative capacity^74–76^. Similarly, our data demonstrated that late stages of cardiomyocyte differentiation are characterized by increased oxidative stress. However, despite the increased ROS, we found no increase in DNA damage (as measured by γ-H2AX foci). Therefore, we suggest that cardiomyocytes at the final stages of differentiation are better suited to survive oxidative environments than their undifferentiated counterparts. Since our in vitro model did not replicate the oxidative event mammalian cardiomyocytes experience after birth, we challenged our cells with H_2_O_2_ to simulate an oxidative event. We found that though cardiomyocytes presented higher levels of ROS they had proportionally less DNA damage than cardiomyoblasts, and had a far superior survival rate. These results corroborated our hypothesis that cardimyocytes have a higher tolerance to oxidative stress and are more effective at repairing DNA damage caused by such stress (Fig. 7). These findings show that the differentiated cells employ specific strategies to minimize genomic damage and maintain DNA integrity during the maturation process.

Overall, our data identifies specific metabolic alterations associated with the metabolic shift during cardiomyocyte differentiation which not only corroborate previous findings but help to complete the picture of cellular landscape during this major event^18^. Additionally we found the shift also leads to increased ROS production and enhanced resistance to cell death. Importantly, cardiomyocytes at the late stage of differentiation maintain a decreased number of γ-H2AX foci, indicating a DNA damage repair capacity that mitigates oxidative damage, which may enhance cell survival in an oxygen rich environment after birth. Better understanding the metabolic shift that occurs during cardiomyocyte differentiation and the implications of this event on cell resilience to oxidative stress offers new insights into potential risk factors and preventative care of cardiovascular diseases.

### Limitations of the study

First, while GC-MS is efficient, sensitive and reproducible, its main drawback is the existence of potential metabolite alterations we cannot detect due to the impossibility of making certain compounds volatile^77^. As such, we recognize that the metabolic alterations here described may be only part of all the metabolic alterations that are occurring during cardiomyocyte differentiation. Secondly, H9c2 cardiomyoblasts are widely used in cardiovascular research and have been shown to differentiate into cardiomyocyte^78^. However, even when fully differentiated this cell line lacks sarcomeric structures and consequently do not beat spontaneously^79^. Therefore, we expect that modulation of some metabolites related to cell contraction might not be accurately represented in this model. On the other hand, alternatives such as primary cardiomyocyte culture are much more difficult to use experimentally and presupposes the use of animals, which should be avoided when possible. Another option might be iPS cells, however, these cells require a longer culture time for proper differentiation and pose their own limitations such as genetic instability linked to mutations during reprogramming^80^. As a result, we chose to use H9c2 cells as our model for this study. Finally, as we explained above, the final stage of cardiomyocyte differentiation is associated with a sudden increase in oxygen levels after birth^4–6^. We could not directly explore the effects of the oxidative event on cardiomyocyte development. Therefore, our findings likely best mirror the differentiation process happening in utero. Though we did challenge our cells with H_2_O_2_ (an approximation to an oxidative event like birth), the effects of environmental oxygen levels were not investigated in this study.

## Materials and methods

### H9c2 cell culture and differentiation

H9c2(2 − 1) cells were acquired from the Rio de Janeiro Cell Bank (BCRJ) under the BCRJ code: 0098.

Cardiomyoblasts from the cell lineage H9c2 (ventricular myoblasts from rat embryos, passage ~ 15) were cultivated in DMEM (Dulbecco’s Modified Eagle Medium - D777) supplemented with 1% penicillin/streptomycin, and 10% FBS (Fetal Bovine Serum) at 37 °C with 5% CO_2_ atmosphere in a humidified incubator. Cells were cultivated and expanded in T75 culture flasks. Then 8 × 10^5^ cells were transferred to Petri dishes and cultivated until they reached approximately 80% of confluence. For the differentiation of cardiomyoblasts into cardiomyocytes, FBS concentration was reduced to 1% and 10 nmol L^− 1^ retinoic acid was added to the culture medium (differentiation medium). The medium was changed every 1–2 days. During differentiation, images were acquired to confirm homogeneous morphological alterations throughout the population (**Supplem.** Figure 1). Cells referred to as cardiomyoblasts (CMB) are cells that have achieved the necessary confluence but are not exposed to differentiation medium, they are therefore Day 0 cells. Cells referred to as cardiomyocytes (CM) are cells kept in differentiation medium between 7 and 10 days, as described previously^18,81–83^. For the oxidative stress assay, cells were treated for 24 h with 500 µM H_2_O_2_, diluted in serum-free medium.

### FLIM

Images from living cells were collected in an inverted LSM780-NLO Microscope (Carl Zeiss AG, Germany) using the EC Plan-Neofluar 40x/1.30 Oil DIC objective. Cells were maintained at 37 C° and 5% CO_2_ during acquisition. Excitation light came from a Chameleon Discovery NX (Coherent - USA), which provides 100 fs pulses at 80 MHz, tuned to 760 nm. Backward scattered signal, after a 690 nm SP, was detected in a hybrid detector HPM-100-40 (Becker-Hickl, Germany) connected to a time correlated single-photon counting module, SPC-830 (Becker-Hickl, Germany). Both broad fluorescence, with no filter, and NADH and FAD fluorescence, using 445 ± 45 nm BP and 535 ± 22 nm BP filters, respectively, were detected. In both measurements the total integration time was 120s. Each image is 512 × 512 pixels and to fit the biexponential decay curve model to the photon histogram we selected a bin of 5 pixels. The fitting was done using the SPCImage 8.9 software. The free parameters are the short and long lifetime components and their respective relative contributions (τ_1_, τ_2_, a_1_, e a_2_). By definition a_1_ + a_2_ = 100% and the mean lifetime is τ_m_ = a_1_τ_1_ + a_2_τ_2_. Afterwards, cells cytoplasm was selected and matrices containing each of these parameters were exported, histograms were made for each of them; their mean and standard deviation were also calculated using $\bar{x} = \sum_{i=0}^{N} \frac{x_i}{N}$ and $\sigma^{2} = \sum_{i=0}^{N} \frac{\left(x_i-\bar{x}\right)^{2}}{N}$. The distribution curve of these parameters in the cytoplasm region was compared between the experimental groups and their difference was quantified with Cohen’s d. The mean Fluorescence-Lifetime Redox Ratio (FLIRR), defined as FLIRR = NADH a_2_ / FAD a_1_, was also calculated in the cytoplasm region of each cell. Similar parameters are reported in literature^84–88^.

### Immunofluorescence assays

Cardiomyoblasts were cultivated on round glass cover slips and divided in four groups: cardiomyoblasts (undifferentiated H9c2 cells; day 0), and differentiating cardiomyocytes for 3, 5, 7, and 10 days. To analyze the morphological alterations of cardiomyocytes during differentiation, cells were fixed with 4% paraformaldehyde, permeabilized in 0.8% Triton X-100 solution and blocked with 5% BSA. Subsequently, cells were incubated with anti-Ki67 antibody. After rinsing, cells were incubated with Alexa Fluor-488 (Thermo Fisher, MA3-96500-A488–1:200) and Alexa 647-Phalloidin-(1:1000; Sigma, P-1951), and 1.,5 µMDAPI (Invitrogen, D1306), for 30 min. In order to study mitochondrial morphology, cells were labeled with 250nM Mitotracker Deep Red (Invitrogen, M22426) for 30 min, then fixed with 4% paraformaldehyde. To study reactive oxygen species production, cells were labeled with 5µM CellRox Green (Invitrogen, C10444) for 30 min and then fixed with 4% paraformaldehyde. Additionally, cells were fixed with 4% paraformaldehyde, permeabilized (0.8% Triton X-100) and blocked (5% BSA) before being incubated with a primary antibody solution for Phospho-Histone H2AX (1:200; Invitrogen, PA5-77995), for 30 min. Then, cells were incubated with an Alexa Fluor secondary antibody (1:2000; Invitrogen, A11008), and 1.5 µM DAPI (Invitrogen, D1306) for 30 min.

Fluorescence images of CellRox and Phalloidin/phospho-H2AX/Dapi were acquired by Superresolution Structured Illumination Microscopy (SR-SIM), in a Zeiss Elyra microscope using a Plan-Apochromat 63x/1.4 Oil DIC objective and EC Plan-Neofluar 10x/0.30 Dry (WD: 5.2 mm) objective for panoramic images. Fluorescence images of MitoTracker were acquired in the Zeiss Airyscan microscope using a C Plan-Apochromat 63x/1.4 Oil DIC (WD:0.14 mm) objective. Acquired images were processed by the microscope’s proprietary Zen software and they were later analyzed using ImageJ.

### Image analysis

For mitochondrial count per cell and morphology analysis, including area, perimeter, form factor (*inverse of circularity perimeter*^*2*^/(4π*area)), aspect ratio (*the ratio of the major axis length to the minor axis length*) and number of branches, we adapted an image analysis pipeline from Mitochondria Analyser plugin^89^.

To analyze the expression of Ki67 we counted the nuclei that were more than 30% covered with Ki67 and normalized by the total number of nuclei obtained on panoramic images. This was done using *thresholding* commands and analyzing the area and intensity of the DAPI and Ki67 staining in ImageJ^89^.

### Sample Preparation

**Fig. 8:**
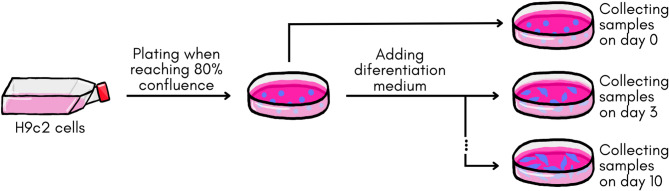
Procedure for cultivating and differentiating H9c2 cells.

H9c2 cardiomyoblasts were cultivated until reaching 80% confluence and, subsequently, the differentiation was induced by incubating the cells with the differentiation medium. The samples were collected on day 0 (undifferentiated cells) and on days 3, 5, 7, and 10 after the addition of the differentiation medium (Fig. 8).

### Cell extraction

**Fig. 9:**
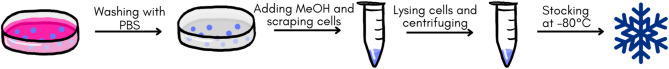
Flowchart of sample preparation for metabolomics analyses. The intracellular metabolites of H9c2 cardiomyocytes were extracted on methanol by freeze-thaw cycles.

To obtain the endometabolome content, the cells were scraped on days 0, 3, 5, 7, and 10 with a rubber cell scraper, and suspended in 800 µL of ice-cold methanol (MeOH) extraction solvent (Fig. 9). Afterward, cells were subject to three freeze-thaw cycles for lysis. Samples were placed in liquid nitrogen for 10 min for rapid freezing and thawed in an ice bath for 10 min. The supernatant was recovered by centrifugation at 5,725 ×g for 5 min at 4 °C. The cell pellet was extracted once more with 400 µL of MeOH. The final supernatant was combined with the previously obtained one. For quality control (QCs) preparation, 100 µL from each sample were pooled together and separated into equal volumes. All samples and QCs were stored at -80 °C until the derivatization step and GC analysis^90^. Triplicates samples were prepared for each condition.

### GC-MS analysis

Samples were thawed on ice and 200 µL was concentrated using a Concentrator plus/Vacufuge^®^ plus (Eppendorf) for 2 h. For derivatization, 20 µL of methoxyamine hydroxychloride (CH_3_ONH_2_∙HCl) in pyridine at a concentration of 15 g L^− 1^ were added to protect the aldehyde and ketone groups by methoximation reaction. Samples were mixed in vortex for 2 min and sonicated for 10 min. Samples were kept in the dark for 16 h. Then, 20 µL of N, O-bis(trimethylsilyl)trifluoroacetamide (BSTFA) with 1% trimethylchlorosilane (TMCS) was added to promote silylation reaction. The final solution was heated at 70 °C for 1 h in an oven.

GC-MS analyses were performed in the Agilent 5975 C equipment with a single quadrupole mass analyzer. An HP-5MS column (30 m x 0.25 mm x 0.25 μm) with low polarity stationary phase composed of 5% phenyl and 95% dimethylpolysiloxane (Agilent Technologies) was used. One µL of the sample was injected using a split/splitless injector operated in split mode (10:1 ratio) using helium as the carrier gas. The initial oven temperature was set at 60 °C (1 min) with a temperature ramp of 10 °C min^− 1^ until the final temperature of 325 °C. Transfer line was maintained at 290 °C, and quadrupole temperature at 150 °C. The mass scanning in the mass spectrometer was performed from 50.0 to 600.0 Da. Agilent Fiehn GC-MS library was used for the identification of the metabolites^91^.

### Data processing and statistical analysis

The chromatograms were processed using MS-Dial software version 4.80 (RIKEN PRIMe)^92^. After peak picking, deconvolution, and alignment, a metabolic feature table was created and then processed in Excel. To reduce uninformative metabolic features, data filtering was performed. Metabolic features with 65% of missing values were removed as well as features with relative standard deviation (RSD) of 30% or more on the QCs. Further data pre-treatment was performed with MetaboAnalyst 5.0, using median normalization, square root transformation, and auto-scaling. Unsupervised and supervised multivariate analyses (PCA and PLS-DA, respectively) were applied to check samples’ clustering trends and discrimination between groups. Variable Importance in Projection (VIP) scores were used to determine significant features for group separation, while unpaired t-Tests with false discovery rate (FDR) were used to analyze the features. p-value < 0.05 indicated statistical difference. From the PLS-DA model, cross-validation was performed to evaluate the R² (goodness of fit) and Q² (goodness of prediction) values.

### Pathway analysis and biological interpretation

**Fig. 10:**
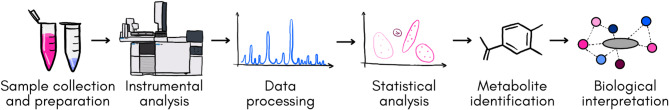
Metabolomics experiment workflow.

Statistically significant molecular features, i.e., FDR < 0.05, p-value < 0.05 and VIP score > 1.0, were annotated using the Automated Mass Spectral Deconvolution and Identification System (AMDIS) with NIST Mass Spectral Library and Agilent Fiehn GC-MS Metabolomics RTL Library based on spectral and retention time matching. With the identification, MetaboAnalyst 5.0 Pathway Analysis tool provided the impact on metabolic pathways, allowing a connection between alterations in the concentration of the compounds found and their role in the process of cell differentiation. Human Metabolome Database (HMDB)^93^, Small Molecule Pathway Database (SMPDB)^94^ and Kyoto Encyclopedia of Genes and Genome (KEGG)^95^, along with the literature, were used to verify how metabolic pathways are modulating during the cardiomyocyte differentiation. Due to metabolites that cannot become volatile and analyzed by GC-MS, we considered that the metabolites found represent only a part of all the altered metabolites (p-value < 0.05) and we applied a more lenient p-value of 0.1 to the pathway enrichment analysis in an effort to compensate for the potential lack of measured metabolite alterations in the pathways, as reported previously^27^. The workflow presented in Fig. 10 summarizes the main steps of the metabolomics experiment.

## Supplementary Information

Below is the link to the electronic supplementary material.

Supplementary Material 1

## Data Availability

The metabolomics dataset generated and analysed during the current study is available in the Metabolomics Workbench repository. [http://dx.doi.org/10.21228/M8NN9R](http:/dx.doi.org/10.21228/M8NN9R)All other data generated and analysed during the current study will be available in the Unicamp Institutional Research Data Repository (REDU).
