# Supplementary material for: Metabolic reprogramming enhances oxidative stress resistance in differentiating cardiomyocytes
Source: Sci Rep. 2026 Jan 20;16:5534. doi: 10.1038/s41598-026-35263-5 (PMC12887050; doi:10.1038/s41598-026-35263-5)
Supplement: Supplementary file 1 — Supplementary Material 1 [file 41598_2026_35263_MOESM1_ESM.docx]

**Metabolic Reprogramming Enhances Oxidative Stress Resistance in Differentiating Cardiomyocytes**

Lara Basseres Novais¹*, Beatriz Rocha Ilidio Rodrigues¹*, Flávia Oliveira Borges Pereira¹, Alan Gonçalves Amaral², Sofya Castilho Lapa¹, Lucas Lopes Maldonado^3^, Pedro Víctor-Carvalho¹, Isabela Aparecida Moretto¹, Hans Rolando Zamora Obando², Mariana Conceição da Silva¹, Ana Paula Samogim¹, Ingridi Rafaela de Brito¹, Maria das Graças de Souza Carvalho¹, Antonio Thiago Pereira Campos^4,5^, Michelle Bueno de Moura Pereira Antunes^6^, Carlos Lenz Cesar^4,5^, Hernandes F. Carvalho^1,4^, Ana Valéria Colnaghi Simionato^2,7^**, André Alexandre de Thomaz^3,4^**, Aline Mara dos Santos^1,4^**.

**
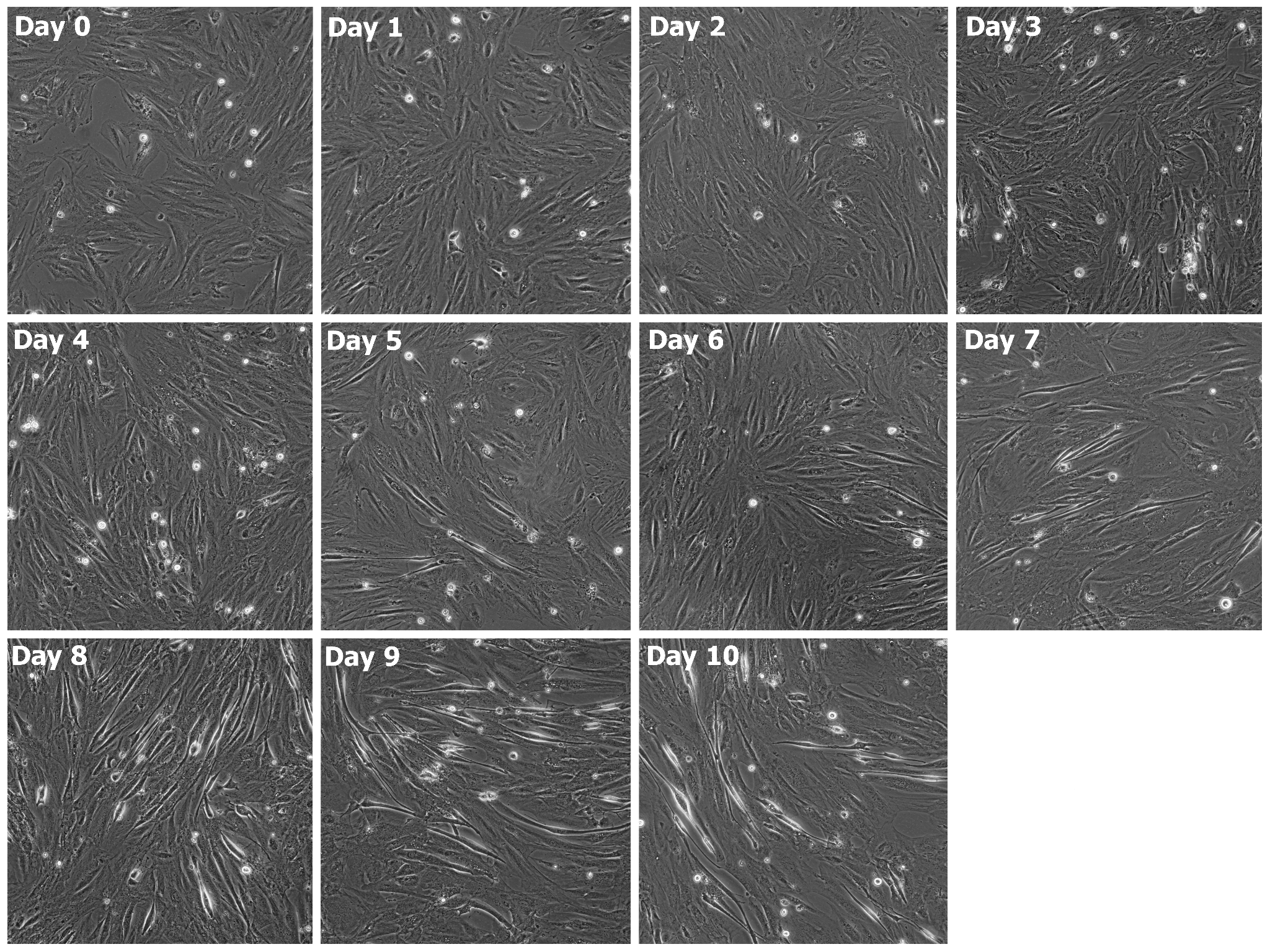
**

**Supplementary Figure 1.** Transmission images of H9c2 cells before (Day 0) and after the differentiation induction (Day 1 to Day 10).

**
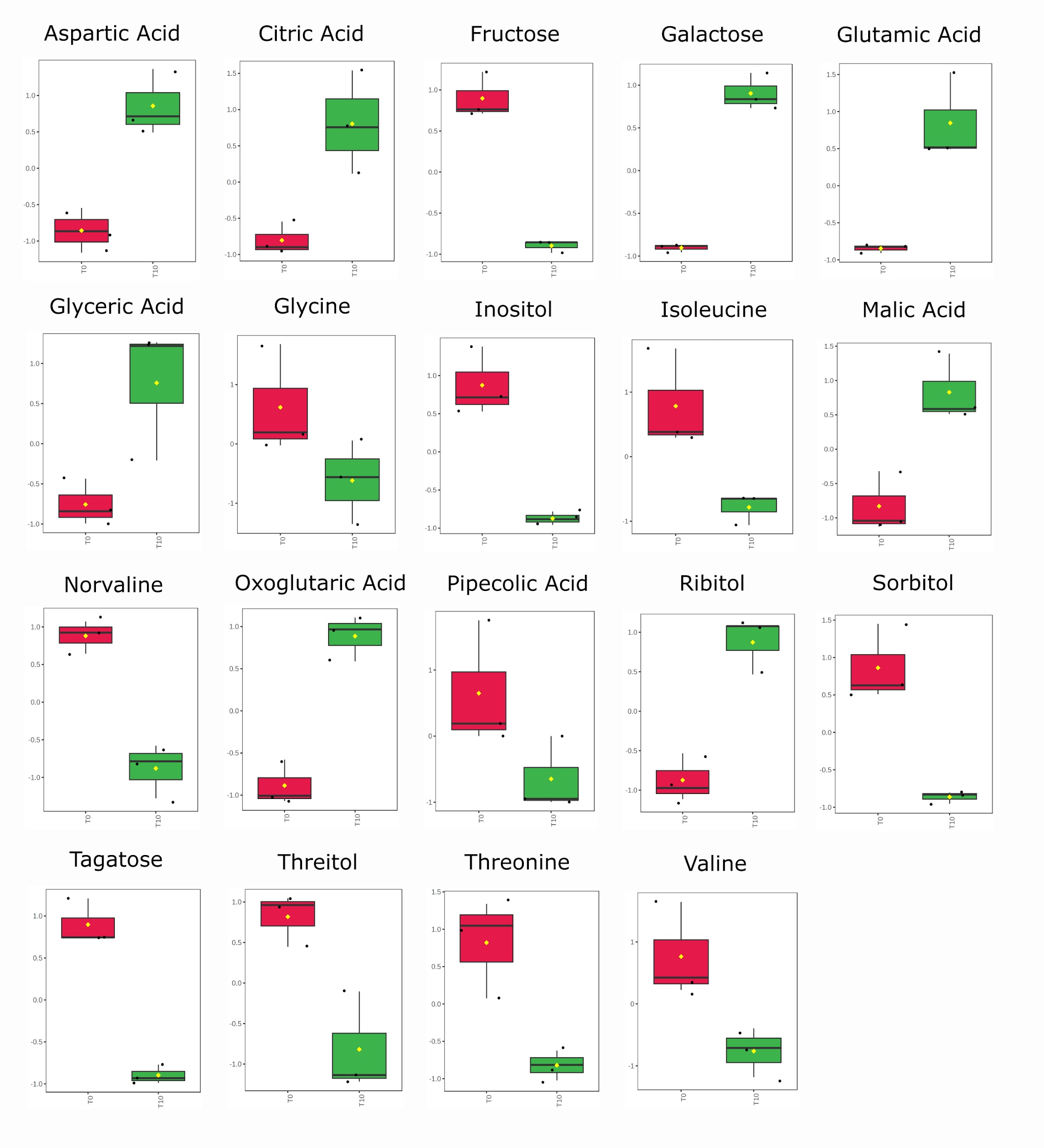
**

**Supplementary Figure 2.** Boxplots of the features shown in the highlighted metabolic pathways, as indicated.

**Supplementary Table 1.** Statistically significant pathways (p value < 1e-01) altered in H9c2 cells after the differentiation process.

| **Pathway** | | | | | |
| --- | --- | --- | --- | --- | --- |
| **Significant metabolites** | **Pathway**  **p value** | **FDR** | **Hits/Total** | **Metabolites up** | **Metabolites down** |
| **Malate-Aspartate Shuttle** | | | | | |
| Glutamic acid ↑, L-Aspartic acid ↑, Oxoglutaric acid ↑, Malic acid ↑ | 1.12e-05 | 8.89e-04 | 4/10 | 4 | 0 |
| **Galactose Metabolism** | | | | | |
| D-Glucose ↓, D-Galactose ↑, D-Mannose ↓, myo-Inositol ↓, Sorbitol ↓, D-Fructose ↓ | 1.81e-05 | 8.89e-04 | 6/38 | 1 | 5 |
| **Glucose-Alanine Cycle** | | | | | |
| D-Glucose ↓, Glutamic acid ↑, Oxoglutaric acid ↑ | 1.05e-03 | 3.31e-02 | 3/13 | 2 | 1 |
| **Ammonia Recycling** | | | | | |
| Glycine ↓, Glutamic acid ↑, L-Aspartic acid ↑, Oxoglutaric acid ↑ | 1.35e-03 | 3.31e-02 | 4/31 | 3 | 1 |
| **Warburg Effect** | | | | | |
| Citric acid ↑, D-Glucose ↓, Glutamic acid ↑, Malic acid ↑, Oxoglutaric acid ↑ | 1.83e-03 | 3.34e-02 | 5/57 | 4 | 1 |
| **Glycine and Serine Metabolism** | | | | | |
| Glycine ↓, Glyceric acid ↑, Glutamic acid ↑, L-Threonine ↓, Oxoglutaric acid ↑ | 2.14e-03 | 3.34e-02 | 5/59 | 3 | 2 |
| **Alanine Metabolism** | | | | | |
| Glycine ↓, Glutamic acid ↑, Oxoglutaric ↑ | 2.39e-03 | 3.34e-02 | 3/17 | 2 | 1 |
| **Transfer of Acetyl Groups into Mitochondria** | | | | | |
| Citric acid ↑, D-Glucose ↓, Malic acid ↑ | 5.12e-03 | 6.28e-02 | 3/22 | 2 | 1 |
| **Glutamate Metabolism** | | | | | |
| Glycine ↓, Glutamic acid ↑, L-Aspartic acid ↑, Oxoglutaric acid ↑ | 6.97e-03 | 7.59e-02 | 4/48 | 3 | 1 |
| **Lactose Degradation** | | | | | |
| D-Glucose ↓, D-Galactose ↑ | 9.10e-03 | 8.28e-02 | 2/9 | 1 | 1 |
| **Arginine and Proline Metabolism** | | | | | |
| Glycine ↓, Glutamic acid ↑, L-Aspartic acid ↑, Oxoglutaric acid ↑ | 9.29e-03 | 8.28e-02 | 4/52 | 3 | 1 |
| **Urea Cycle** | | | | | |
| Glutamic acid ↑, L-Aspartic acid ↑, Oxoglutaric ↑ | 1.02e-02 | 8.35e-02 | 3/28 | 3 | 0 |
| **Fructose and Mannose Degradation** | | | | | |
| D-Mannose ↓, Sorbitol ↓, D-Fructose ↓ | 1.36e-02 | 9.70e-02 | 3/31 | 0 | 3 |
| **Valine, Leucine and Isoleucine Degradation** | | | | | |
| Glutamic acid ↑, Isoleucine ↓, Oxoglutaric acid ↑, L-Valine ↓ | 1.45e-02 | 9.70e-02 | 4/59 | 2 | 2 |
| **Citric Acid Cycle** | | | | | |
| Citric acid ↑, Malic acid ↑, Oxoglutaric acid ↑ | 1.48e-02 | 9.70e-02 | 3/32 | 3 | 0 |
| **Gluconeogenesis** | | | | | |
| D-Glucose ↓, Oxoglutaric acid ↑, Malic acid ↑ | 1.62e-02 | 9.70e-02 | 3/33 | 2 | 1 |
| **Beta-Alanine Metabolism** | | | | | |
| Glutamic acid ↑, L-Aspartic ↑, Oxoglutaric acid ↑ | 1.75e-02 | 1.01e-01 | 3/34 | 3 | 0 |
| **Aspartate Metabolism** | | | | | |
| Glutamic acid ↑, L-Aspartic acid ↑, Oxoglutaric ↑ | 1.90e-02 | 1.03e-01 | 3/35 | 3 | 0 |
| **Tyrosine Metabolism** | | | | | |
| Dopamine ↑, Glutamic acid ↑, L-Aspartic acid ↑, Oxoglutaric acid ↑ | 2.60e-02 | 1.34e-01 | 4/70 | 4 | 0 |
| **Propanoate Metabolism** | | | | | |
| Glutamic acid ↑, Oxoglutaric acid ↑, L-Valine ↓ | 3.09e-02 | 1.51e-01 | 3/42 | 2 | 1 |
| **Glutathione Metabolism** | | | | | |
| Glycine ↓, Glutamic acid ↑ | 4.30e-02 | 2.01e-01 | 2/20 | 1 | 1 |
| **Carnitine Synthesis** | | | | | |
| Glycine ↓, Oxoglutaric acid ↑ | 5.13e-02 | 2.28e-01 | 2/22 | 1 | 1 |
| **Cysteine Metabolism** | | | | | |
| Glutamic acid ↑, Oxoglutaric acid ↑ | 6.94e-02 | 2.28e-01 | 2/26 | 2 | 0 |
| **Phenylalanine and Tyrosine Metabolism** | | | | | |
| Glutamic acid ↑, Oxoglutaric acid ↑ | 7.42e-02 | 3.03e-01 | 2/27 | 2 | 0 |
| **Lysine Degradation** | | | | | |
| Glutamic acid ↑, Oxoglutaric acid ↑ | 8.92e-02 | 3.50e-01 | 2/30 | 2 | 0 |
| **Amino Sugar Metabolism** | | | | | |
| Glutamic acid ↑, D-Fructose ↓ | 1.05e-01 | 3.96e-01 | 2/33 | 1 | 1 |


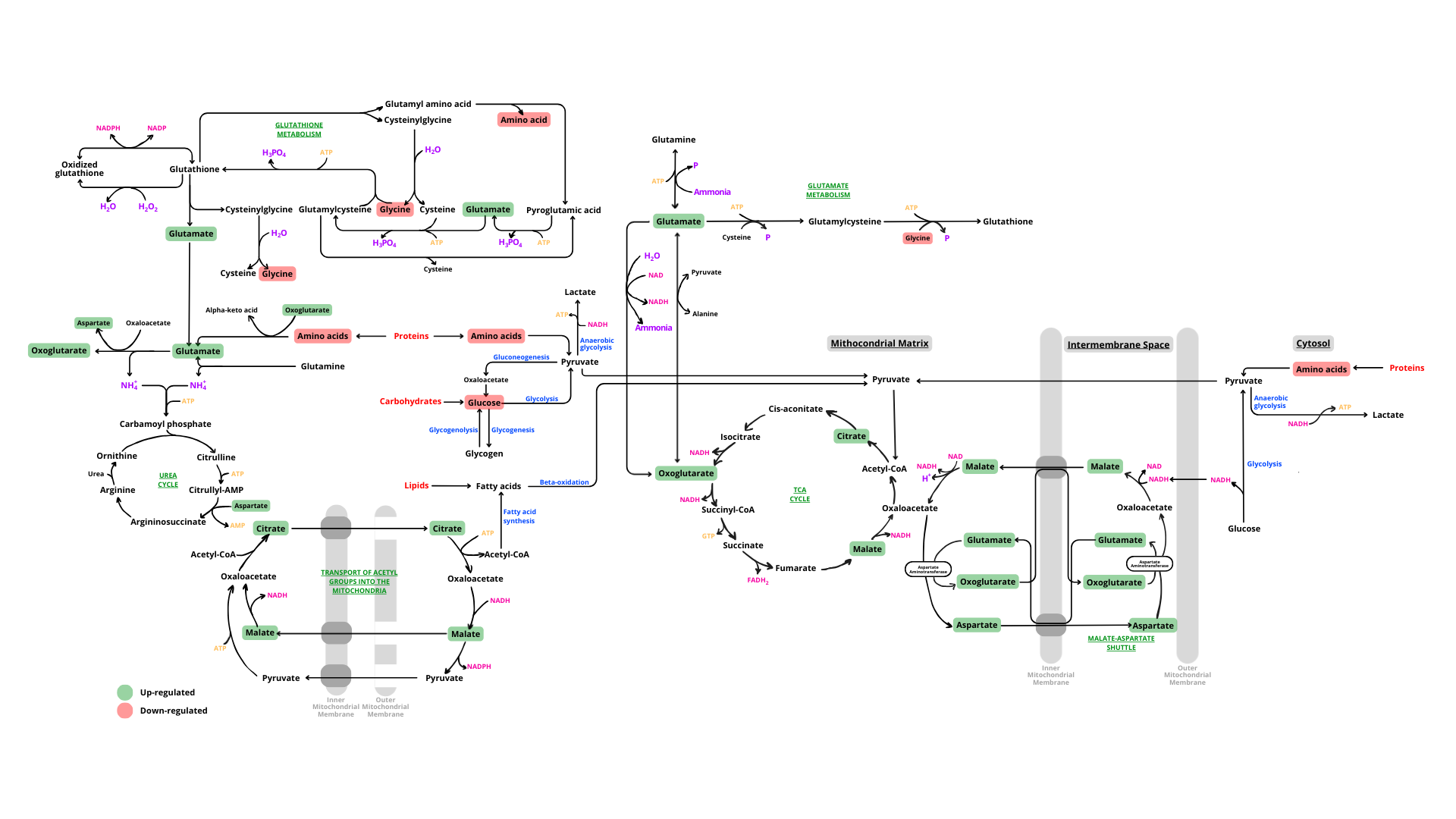


**Supplementary Figure 3.** Overview of significant metabolic pathways and their links.

**
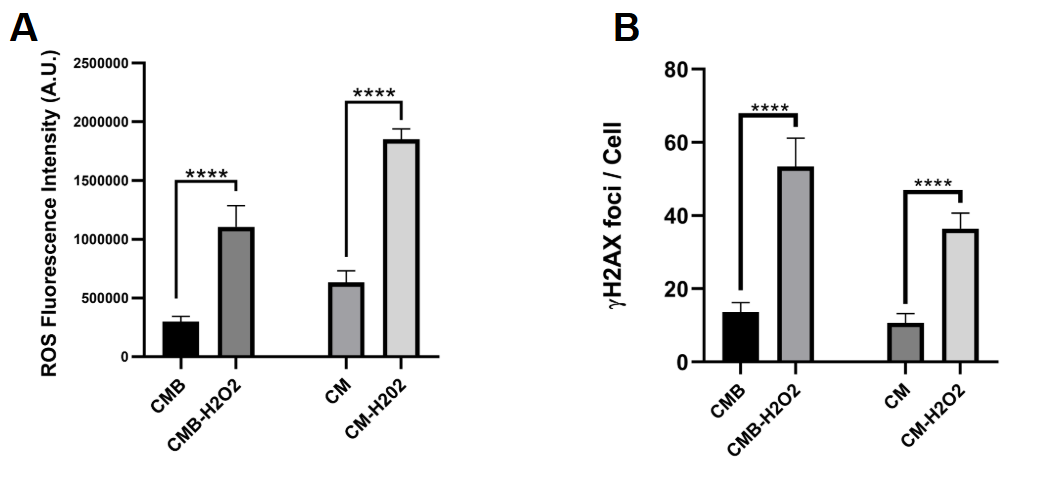
Supplementary Figure 4.** Peroxide treatment was effective in increasing ROS and DNA breaks in both cardiomyoblasts and cardiomyocytes. **A.** Graph showing increase in ROS in both CMB and CM treated with peroxide. Data are represented by means ± SEM. Kolmogorov-Smirnov test was used to assess normality and Test t or Mann-Whitney test were used to compare the groups; *p ≤ 0.05; ***p < 0.001. **B.** Graph showing increase in γ-H2AX foci in both CMB and CM treated with peroxide. Data are represented by means ± SEM. Kolmogorov-Smirnov test was used to assess normality and Test t or Mann-Whitney test were used to compare the groups; *p ≤ 0.05; ***p < 0.001.

**
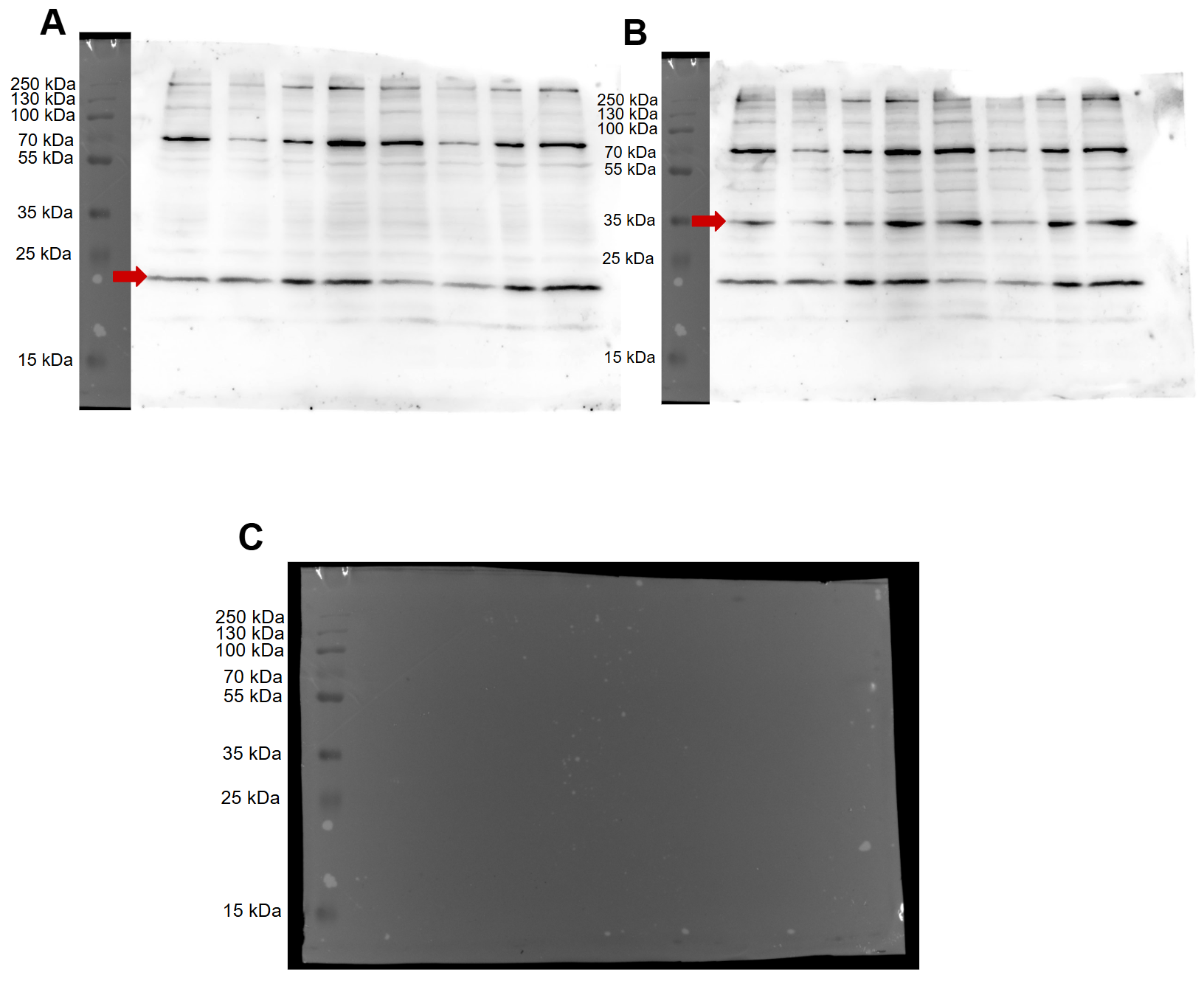
**

**Supplementary Figure 5.** Uncropped blots from Figure 7. **A.** Blot of PUMA, indicated by the red arrow. **B.** Blot of GAPDH, indicated by the red arrow. **C.** Uncropped image of the membrane showing the ladder.
